# A Directed RNAi Screen Based on Larval Growth Arrest Reveals New Modifiers of *C. elegans* Insulin Signaling

**DOI:** 10.1371/journal.pone.0034507

**Published:** 2012-04-12

**Authors:** Ola Billing, Balasubramanian Natarajan, Ateequrrahman Mohammed, Peter Naredi, Gautam Kao

**Affiliations:** Department of Surgical and Perioperative Sciences, Umeå University, Umeå, Sweden; University of North Carolina at Chapel Hill, United States of America

## Abstract

Genes regulating *Caenorhabditis elegans* insulin/IGF signaling (IIS) have largely been identified on the basis of their involvement in dauer development or longevity. A third IIS phenotype is the first larval stage (L1) diapause, which is also influenced by *asna-1*, a regulator of DAF-28/insulin secretion. We reasoned that new regulators of IIS strength might be identified in screens based on the L1 diapause and the *asna-1* phenotype. Eighty- six genes were selected for analysis by virtue of their predicted interaction with ASNA-1 and screened for *asna-1*-like larval arrest. *ykt-6*, *mrps-2*, *mrps-10* and *mrpl-43* were identified as genes which, when inactivated, caused larval arrest without any associated feeding defects. Several tests indicated that IIS strength was weaker and that insulin secretion was defective in these animals. This study highlights the role of the Golgi network and the mitochondria in insulin secretion and provides a new list of genes that modulate IIS in *C. elegans*.

## Introduction

The insulin/IGF signaling (IIS) pathway has been extensively studied in *C. elegans* because it plays a central role in different aspects of the life history of the animal. These roles include dauer entry and exit, life span, innate immunity, heat and oxidative stress responses, and associative learning [1]. Most of the regulators of insulin signaling have largely been found either by looking for genes which when inactivated cause defects in dauer formation or in longevity. In recent years a role for IIS has been demonstrated in the execution of the first larval stage (L1) larval diapause [2], [3]. Larvae enter this diapause if they hatch into an environment that lacks food. They can remain in this state for about two weeks and resume growth when food is supplied again. A strong *daf-2/insulin receptor* mutant or over expression of a *daf-2* antagonist causes worms to arrest in this state without loss in feeding ability. It is noteworthy that mutations in most other genes affecting IIS activity do not display this phenotype perhaps because sufficiently strong alleles do not exist. For instance weaker *daf-2* mutants form dauers, are long lived and do not arrest as L1 larvae. [4], [5].

The ATPase ASNA-1 is required for both growth and the L1 diapause. A strong reduction of *asna-1* activity by the use of injection RNAi causes worms to arrest as L1 larvae, but with the ability to resume growth if gene activity is restored. A weaker interference with ASNA-1 function, by feeding RNAi, results in a slow growth phenotype and leads to arrest at different larval stages. Finally *asna-1* mutants possessing maternal but not zygotic gene activity grow up to become scrawny sterile adults. Notably, even though *asna-1* larvae arrest growth in the presence of food, they do not have feeding defects, suggesting a lack of coordination between growth and nutrient availability. *asna-1* promotes growth non-autonomously and mutants in the gene have severely reduced IIS activity. *asna-1* mutants are defective in DAF-28/insulin secretion which is the likely cause of its growth defect [6]. In some respects the *asna-1* mutant phenotype resembles that of *daf-2* mutants. However, while *daf-2* mutants form dauers at 25°C, *asna-1* mutants only do so in sensitized genetic backgrounds. Therefore *asna-1* would likely not be found in screens for mutants in dauer formation or longevity.

With the understanding of the *asna-1* phenotype as it relates to the IIS, we wished to ask whether new genes affecting IIS strength could be found that, like *asna-1*, primarily had a larval growth phenotype when they were inactivated. We attempted to find such potential modulators of IIS by making a list of predicted interactors of ASNA-1and its homologs in the budding yeast and fruit flies, and then by screening these genes for *asna-1*-like growth defects and early larval arrests after RNAi knockdown. Employing this strategy we have identified genes for three proteins of the mitochondrial ribosome and a Golgi-associated v-SNARE as new modulators of IIS and DAF-28/insulin secretion. We show that knocking down activity of genes encoding mitochondrial ribosomal proteins leads to decreased mitochondrial membrane potential. This may in turn lead to alterations in the ATP/ADP ratio, which is a key stimulator of insulin secretion. In the case of *ykt-6* its demonstrated role in Golgi function is consistent with the notion that in insulin secretion may be reduced because dense core vesicle biogenesis is affected in these animals.

## Materials and Methods

### *C. elegans* strains and maintenance

Worms were maintained under standard conditions [7] at 20°C on NGM plates unless otherwise stated. N2 is the wild type parent for all the strains in the study. The mutants and transgenes used were *rrf-3 (pk1426), daf-7(e1372), daf-2(1370), daf-16(mgDf50), svIs69 (pdaf-28::daf-28::gfp), zcIs13(phsp-6::gfp), arIs37(pmyo-3::ss::gfp), zIs356(pdaf-16::daf-16::gfp)* and *dvIs19 (pgst-4::gfp::NLS)*. All the mutants and transgenic strains are described in wormbase (www.wormbase.org)

### Plasmids

Feeding RNAi plasmids for *mrps-10*, *mrpl-43* and *mrrf-1* were constructed by amplifying the entire genomic coding region and cloning them into L4440 as BglII/NcoI fragments.

### Feeding RNAi

All the bacterial clones expressing dsRNA for the RNAi experiments, except for the ones described above, came from a library that was purchased from the Ahringer lab [8]. Feeding RNAi was performed as described [9]. The *rrf-3 (pk1426)* strain was used for the RNAi experiments unless otherwise stated.

### Pharyngeal pumping assay

RNAi against the genes that was tested for the assay was performed as described above. The number of pharyngeal strokes per 20 seconds of each such RNAi treated animal was counted under a Leica MZFLIII dissecting microscope at 400× magnification. A graph of recordings was plotted using GraphPad Prism version 5.0c (for Macintosh).

### Fluorescent beads uptake assay

Fluoresbrites 0.2 mm fluorescent beads (Polysciences, Inc.) were mixed with RNAi bacterial suspensions at a 1∶50 dilution and seeded onto RNAi plates incubated overnight. The RNAi against the genes that were tested for the assay was performed as described above. The worms thus obtained were incubated on the plates containing beads for 2 hrs. Micrographs were taken using a Leica DMRB microscope equipped with fluorescence optics at 63× magnification.

### FM4-64 dye uptake assay

The RNAi affected worms were incubated in a solution of 0.4 mM FM4-64 dye (Molecular Probes) for 1 hr and the animals were then transferred to M9 buffer and incubated for 1 hr. Micrographs of the treated worms were taken using the fluorescence microscope at 63× magnification.

### DAF-16::GFP assay

The *rrf-3 (pk1426)* was crossed into worms carrying the integrated DAF-16::GFP (*zIs356*) transgene and used for the studies. RNAi against the genes were performed as described in feeding RNAi technique. The worms obtained after RNAi treatment were micrographed using fluorescence microscope at 100× magnification.

### Dauer phenotype enhancement studies

Experiments to check the enhancement of the Daf-c phenotype of *daf-7 (e1372ts)* were done at 20°C and for the *daf-2(e1370ts)* mutant they were done at 15°C. RNAi against each gene in both mutants was performed at least two times. The number of semidauers and dauers formed were counted and a graph was plotted using GraphPad Prism version 5.0c. Dauer larvae were tested for resistance to 1% SDS [10] by placing them in 200 uL of the solution for 10 minutes at room temperature and then transferring them to a seeded NGM plate to assess how many worms were alive. No L3 stage (non-dauer) wild-type larvae, exposed to the 1% SDS solution for 10 minutes in parallel, survived this treatment.

### *phsp-6::GFP* reporter assay

Worms carrying the integrated *phsp-6::GFP* reporter transgene *(zcIs13)* were treated with feeding RNAi bacteria as described in the feeding RNAi section. Micrographs were taken using the fluorescence microscope at 63× magnification.

### TMRE staining

The arrested larvae arising from feeding RNAi against the tested genes were stained with Tetramethylrhodamine, ethyl ester (TMRE) dye [11]. For quantification of the staining, the images from the fluorescence microscope were converted to grayscale and the region of each worm from the beginning of the intestine to the rectum was selected and used for pixel intensity measurements using the ImageJ software.

### Statistics

A mixed-effect model with Poisson errors was used to assess the effects of the *daf-16(mgDf50)* mutation to growth of the seven RNAi treatments. The proportion of individuals reaching above stage L3 was considered as the response variable and addition of the *daf-16* mutation was considered as treatment. RNAi regimens were fixed effects together with their interactions. The significance level of wild type×treatment interaction was calculated using a likelihood-ratio test. The mixed-effects model analysis was carried out using the R 2.14.0 software [12].

## Results

### Identification of Genes that Promote Larval Development

**Figure 1:**
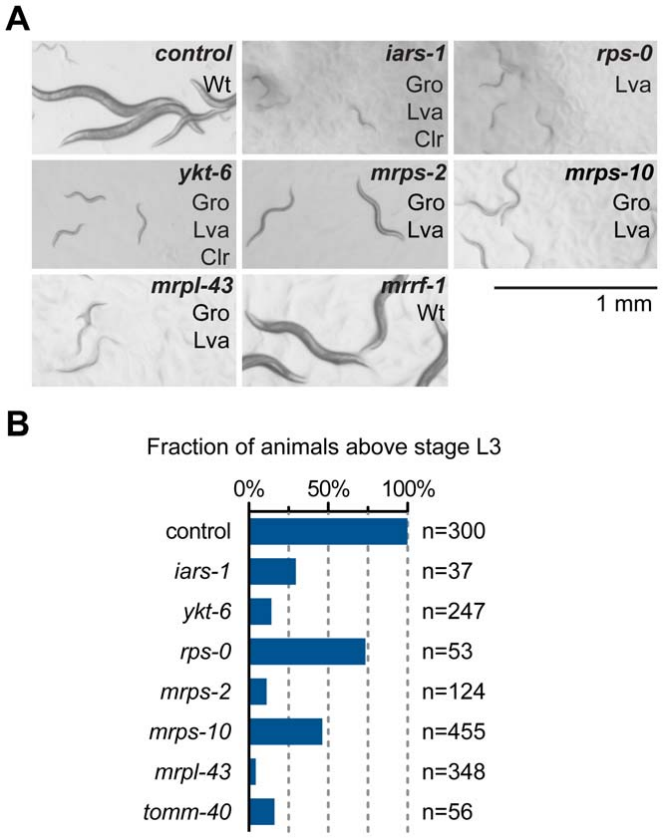
Feeding RNAi analysis of the genes affecting *C. elegans* larval development. A) Feeding RNAi against several genes, but not *mrrf-1*, caused larval growth phenotypes. Gro: Growth arrest; Lva: larval arrest; Clr: Clear body; WT: wild-type. Empty vector (L4440) feeding RNAi was used as a control in all experiments. B) Fraction of animals above stage L3 after RNAi treatments in one representative experiment.

Yeast two hybrid studies, co-immunoprecipitation studies and genome-wide predictions have led to the identification of thirteen genes that interact with *C. elegans asna-1* [13], [14], six genes that interact with the *Drosophila melanogaster* homologue CG1598 [15] and 77 genes that interact with the *Saccharomyces cerevisiae* homologue Get3 [16], [17], [18]. This database of physical interactors is compiled at http://thebiogrid.org. We identified *C. elegans* homologues of the *D. melanogaster* and *S. cerevisiae* interactors using BLASTp and SMART [19] sequence comparison tools. This provided a list of 143 genes of which 86 were tested by feeding RNAi (Table S1) in the RNAi hypersensitive mutant *rrf-3(pk1426)* [20]. In the first stage of the screen we looked for RNAi clones that did not allow the *rrf-3* mutant worms to grow beyond the 3^rd^ larval stage (L3) after 4 days and that produced larvae that were scrawny and pale in appearance (Figure 1A and 1B). This phenotype is similar to the feeding RNAi phenotype of *asna-1* and represents a weaker form of the strict L1 arrest phenotype that is produced only with injection RNAi.

The genes identified in this manner were *mrps-2/mitochondrial ribosomal protein* (T23B12.3), *ykt-6/v-SNARE* (B0361.10), *goa-1*(C26C6.2), F41C3.4/Golgi transport 1 homolog, *rps-0/cytoplasmic ribosomal protein* (B0393.1), *iars-1/isoleucyl tRNA synthetase* (R11A8.6), *enpl-1/GRP94* (T05E11.3), and *tomm-40/translocase of outer mitochondrial membrane* (C18E9.6). The feeding RNAi plasmids were sequenced to ensure that they were correct. We have previously reported a modulatory function for *tomm-40* in IIS activity [21] and the study of *enpl-1* will be described elsewhere (B.N, P.N & G.K in preparation). Analysis of the remaining six genes is described below.

**Figure 2:**
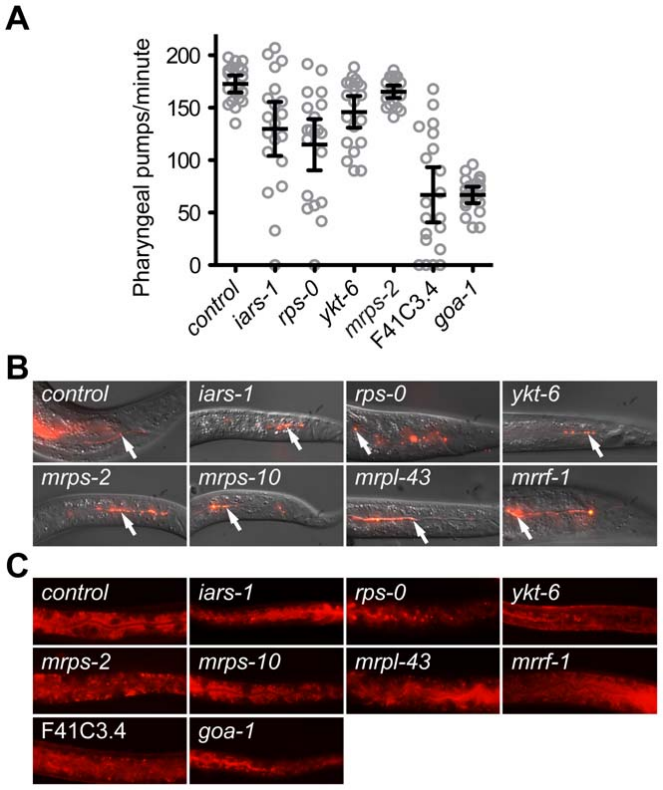
Analysis of pharyngeal pumping, feeding and endocytosis in RNAi treated animals. A) Quantification of pharyngeal pumping rates after feeding RNAi. Each circle represents a single animal. Error bars represent a 95% confidence interval of the mean pumping rate. B) Overlay of differential interference contrast microscopy (DIC) and fluorescence images of worms exposed to RNAi against indicated genes and control RNAi to assay for their ability to ingest fluorescent beads. Arrows indicate accumulation of beads in the intestinal lumen. C) Fluorescence images of worms tested for uptake of FM4-64 dye by intestinal cells after exposure to RNAi against indicated genes and control RNAi.

*asna-1(RNAi)* animals do not display a feeding defect or developmental defects, despite having a growth delay phenotype [6]. To determine which of the genes when knocked down showed this aspect of the *asna-1* phenotype we examined the knockdown animals for feeding defects or developmental abnormalities that might cause growth arrest. Feeding ability was assayed by the pharyngeal pumping rate, ability of worms to ingest bacterial-sized beads and of their intestinal cells to take up luminal contents by endocytosis. Knockdown of *mrps-2*, *ykt-6*, *iars-1* and *rps-0* had little or no effect on pharyngeal pumping (Figure 2A), bead uptake (Figure 2B), or internalization of the dye FM4-64 by intestinal cells in affected animals (Figure 2C). By contrast, pharyngeal pumping was much reduced in *F41C3.4 (RNAi)* and *goa-1(RNAi)* animals (Figure 2A), which raised the possibility that these animals were growth retarded because of starvation induced by pharyngeal defects. Consequently these genes were not analyzed any further. Knockdown of *mrps-2*, *ykt-6*, *iars-1* and *rps-0* did not produce animals with any visible developmental or anatomical defects that would explain the retarded growth. Consistently, the affected animals resumed growth on normal bacteria, indicating that the growth arrest was reversible and not due to catastrophic defects. We next asked whether the phenotypes obtained by down-regulation of *mrps-2* could also be elicited by RNAi against genes encoding two other mitochondrial ribosomal proteins, *mrps-10 (Y37D8A.18)* and *mrpl-43 (C25A1.13)*, and an annotated mitochondrial ribosome recycling factor *mrrf-1 (T20F5.3)* (www.wormbase.org). However, since *mrrf-1* does not have a mitochondrial targeting sequence (Table S2) it may not function in the mitochondria. A retarded growth phenotype was observed upon inactivation of *mrps-10* and *mrpl-43*, but not *mrrf-1* (Figure 1A). *mrps-10(RNAi)* and *mrpl-43(RNAi)* animals did not display any defects in feeding ability (Figure 2A, 2B and 2C). Taken together the results show that *mrps-2, mrps-10, mrpl-43, ykt-6, iars-1* and *rps-0* are required for larval growth but not for feeding and were possible modulators of IIS.

### Analysis of Insulin/Igf Activity in the Growth Defective Animals

We next asked whether the reversible growth arrest in the presence of food and in the absence of feeding defects was due to defective IIS activity. To do this three well characterized assays were used to examine the strength of the IIS pathway *in vivo*: First, when IIS is weak, localization of the downstream transcription factor DAF-16 is nuclear and not cytoplasmic. This is assayed by using a functional *daf-16::gfp* reporter [22]. Second, the ability to form dauers or semi-dauers under conditions that normally prevent dauer formation indicates that the activity of the IIS or TGF*β* pathways may be weak. If inactivation of a candidate gene is synergistic for dauer formation with a weak TGF*β* pathway mutation [23], [24] but not with a weak insulin pathway mutation, this would indicate that RNAi against the candidate gene likely causes reduced IIS. Third, if the larval growth phenotype is caused by weak IIS, then it should be suppressed in a *daf-16* null mutant because *daf-16* is the most downstream negative regulator of this pathway.

**Figure 3:**
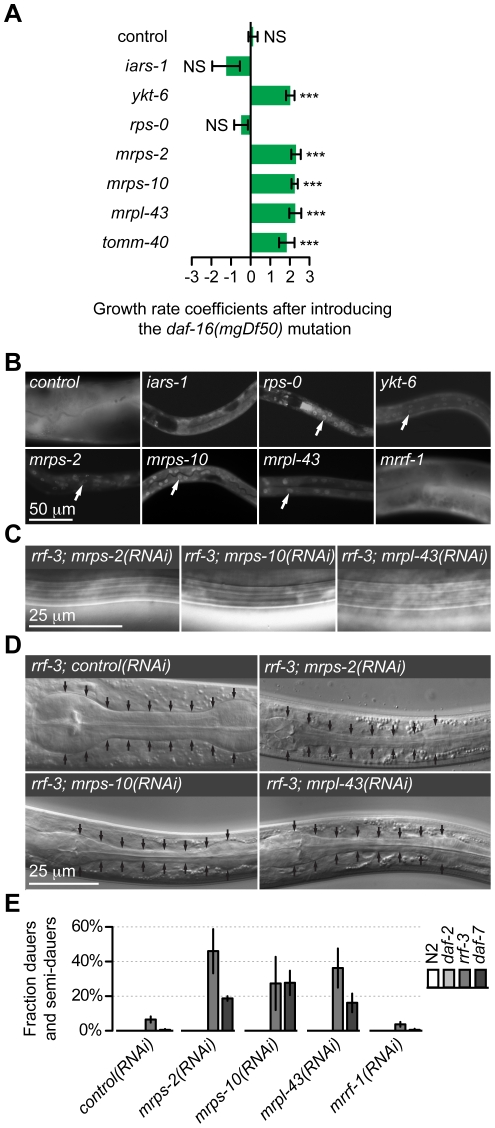
Effect of depletion of gene function of identified genes on insulin/IGF signaling. A) The growth defect by RNAi against *ykt-6*, *mrps-2*, *mrps-10*, *mrpl-43* and *tomm-40* is suppressed in *daf-16* mutants. Bar plot showing mean growth rate coefficients between wild-type and *daf-16(mgDf50)* animals after RNAi. 0 corresponds to no difference in growth above stage L3 between *daf-16(mgDf50)* mutants and wild-type animals. Positive values indicate better growth in the *daf-16* background, where 1 corresponds to a twice as large fraction growing past stage L3 in RNAi treated *daf-16* worms vs. RNAi treated wild-type worms. 2 corresponds to a three times as large fraction growing past stage L3 etc. Error bars are +/− SE for two individual experiments. NS = not significant, *** P<0.001. Significance levels were calculated using *z*-tests. B) Fluorescence micrographs showing nuclear/cytoplasmic localization of DAF-16::GFP after RNAi against indicated genes. Arrows indicate nuclei that accumulate DAF-16::GFP. C) DIC micrographs showing dauer-specific alae in dauer larvae with the indicated RNAi treatments. D) DIC micrographs showing radially constricted pharynges in dauer larva with the indicated RNAi treatments compared to a control larva in stage L3. Arrows indicate outline the region between the two pharyngeal bulbs. E) Quantification of dauer formation in animals with RNAi against the indicated genes in wild-type, *daf-2*, *rrf-3*, and *daf-7* mutants in two experiments. **Control (RNAi):** N2(n = 600), *daf-2*(n = 220), *rrf-3*(n = 660), *daf-7*(n = 655). ***mrps-2(RNAi)*** **:** N2(n = 600), *daf-2*(n = 197), *rrf-3*(n = 640), *daf-7*(n = 630). ***mrps-10(RNAi)*** **:** N2(n = 600), *daf-2*(n = 202), *rrf-3*(n = 920), *daf-7*(n = 600). ***mrpl-43(RNAi)*** **:** N2(n = 600), *daf-2*(n = 189), *rrf-3*(n = 730), *daf-7*(n = 615). ***mrrf-1(RNAi)*** **:**N2(n = 600), *daf-2*(n = 189), *rrf-3*(n = 980), *daf-7*(n = 753). The N numbers are the total of two experiments. Error bars are +/− SE for two individual experiments.

With the exception of *iars-1*, RNAi against all genes that produced a nutrient insensitive retarded growth phenotype also led to the nuclear localization DAF-16::GFP (Figure 3B). The nuclear localization of DAF-16::GFP indicated that it was likely that IIS strength might be lower in these animals. However several other signaling pathways and metabolic conditions also alter the localization of DAF-16::GFP, so this finding on its own suggested, but did not confirm that IIS activity was reduced.

RNAi against *mrps-2*, *mrps-10* and *mrpl-43* in the RNAi hypersensitive *rrf-3* background led to production of a large number of complete dauers that had all the dauer characteristics [10], including elaboration of dauer alae (Figure 3C), dauer specific constricted pharynges (Figure 3D) and SDS resistance of the larvae. Specifically, 48/50 *mrps-2(RNAi)* dauers, 56/58 *mrps-10(RNAi)* dauers, and 62/64 *mrpl-43(RNAi)* dauers were resistant to 1% SDS. RNAi against *mrps-2*, *mrps-10* and *mrpl-43* in *rrf-3(+)* animals did not lead to dauer larvae production, but this treatment enhanced the temperature sensitive dauer constitutive (Daf-c) phenotype of heat sensitive *daf-7/TGFβ* mutant animals at 15°C. However the same RNAi treatment did not enhance the Daf-c phenotype of heat sensitive *daf-2/insulin receptor* mutants (Figure 3E). This differential ability of the RNAi treatments to enhance mutants in the TGF-β signaling pathway but not those in the IIS pathway shows that *mrps-2*, *mrps-10* and *mrpl-43* down-regulation likely modulates the IIS pathway. To investigate this notion further we next tested whether growth was dependent on DAF-16 function.

DAF-16/FOXO is the most downstream component of the IIS and it is negatively regulated by high IIS activity. Consequently IIS is constitutively high in *daf-16* null mutants [25]. We expected that if growth arrest is due to low IIS activity upon knockdown of the genes under study, this phenotype should be suppressed by the high IIS activity in *daf-16* mutants. We observed that the larval growth phenotype of *mrps-2, mrps-10, mrpl-43* and *ykt-6* RNAi knockdown worms was significantly suppressed in the strong *daf-16(mgDf50)* mutant, while loss of *daf-16* had no effect on the growth phenotype of *rps-0* and *iars-1* knockdown animals (Figure 3A). We have previously reported that *tomm-40(RNAi)* also reduces IIS strength and produces a similar larval growth phenotype due to defective IIS strength [21]. As a control, the larval growth phenotype was examined in *tomm-40(RNAi);daf-16(mgDf50)* animals. Here too the growth defect was significantly suppressed. In all cases, RNAi was also performed in parallel in wild-type animals where, as before, larval arrest was observed indicating that the RNAi regime was effective and that the growth suppression was due to the *daf-16* mutation.

Thus the growth arrest observed upon down-regulation of *mrps-2*, *mrps-10*, *mrpl-43* and *ykt-6*, but not that of *iars-1* and *rps-0* requires the activity of DAF-16. Taken together, our studies show that *mrps-2*, *mrps-10*, *mrpl-43* and *ykt-6* positively modulate IIS pathway activity and this modulation is dependent on DAF-16 function. Although *rps-0 (RNAi)* and *iars-1 (RNAi)* displayed a strong growth arrest phenotype and *rps-0 (RNAi)* animals had nuclear localized DAF-16::GFP, the larval arrests were not *daf-16* dependent and knockdown animals did not form dauers or semi-dauer larvae. These findings suggest that the larval growth phenotype seen upon RNAi of *iars-1* and *rps-0* is not due to a reduced IIS activity.

### Low Insulin/IGF Signaling Activity Is Associated with Mitochondrial Dysfunction

**Figure 4:**
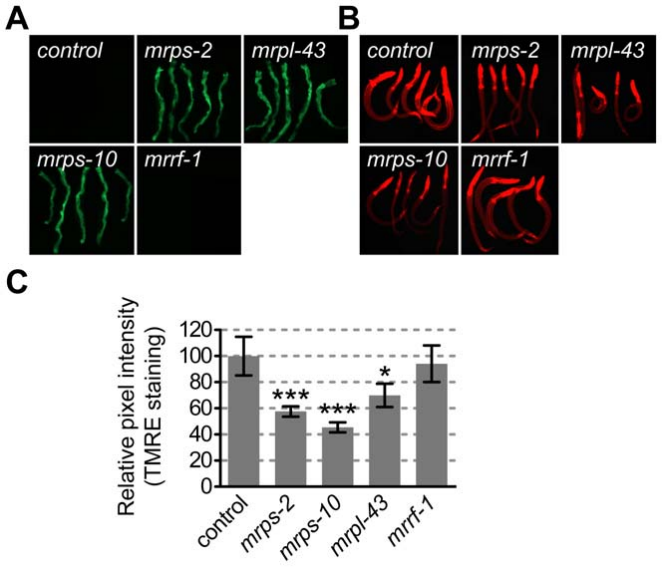
Analysis of mitochondrial function after RNAi against mitochondrial ribosomal protein encoding genes. A) Fluorescence micrographs showing induction of phsp-6::GFP after RNAi against the indicated genes. Five animals are displayed in each panel. B) Fluorescence micrographs depicting TMRE staining in animals after RNAi against the indicated genes. Five animals are displayed in each panel. C) Mean TMRE fluorescence intensities relative to the control in RNAi-treated animals. Control animals were fed with empty vector L4440. Error bars represent a 95% confidence interval of the mean fluorescence intensity. Significance levels were calculated by student's t-tests vs. control. *** P<0.0001 and * P = 0.017.

Three genes identified in the screen are predicted with high confidence (Table S2) to be mitochondrial proteins. To understand how they might affect IIS, we investigated whether RNAi against these genes caused mitochondrial dysfunction. A mitochondrial specific stress response is evoked and *phsp-6::gfp* is induced when the mitochondrial milieu is substantially degraded [11]. *phsp-6::gfp* was induced after knockdown of *mrps-2, mrps-10* and *mrpl-43* genes, but not *mrrf-1* (Figure 4A). Mitochondrial dysfunction was also tested by assessing levels of the mitochondrial proton gradient in animals stained with the dye TMRE [11]. *mrps-2, mrps-10* and *mrpl-43 (RNAi)* animals had reduced TMRE staining in the intestinal region, while control and *mrrf-1(RNAi)* had a consistent staining pattern throughout the animals (Figure 4B). Quantification of the TMRE staining (Figure 4C) shows that staining in *mrps-2(RNAi)*, *mrps-10(RNAi)* and *mrpl-43(RNAi)* animals were significantly different from that seen in the control RNAi animals. This indicated that the proton gradient was weaker in animals with knockdown of *mrps-2*, *mrps-10* and *mrpl-43* due to a diminished flow of electrons through the electron transport chain, while this was not the case in *mrrf-1(RNAi)* animals. The induction of mitochondrial stress appeared to be specific because we did not detect any increase in oxidative stress using the *gst-4::gfp* reporter in these animals (Figure S2). Taken together, the results show that the RNAi treatment against *mrps-2*, *mrps-10* and *mrpl-43* caused significant levels of mitochondrial dysfunction and suggest that the weak IIS may be attributable to this defect.

### Insulin Secretion Is Defective in the Growth Defective Animals

**Figure 5:**
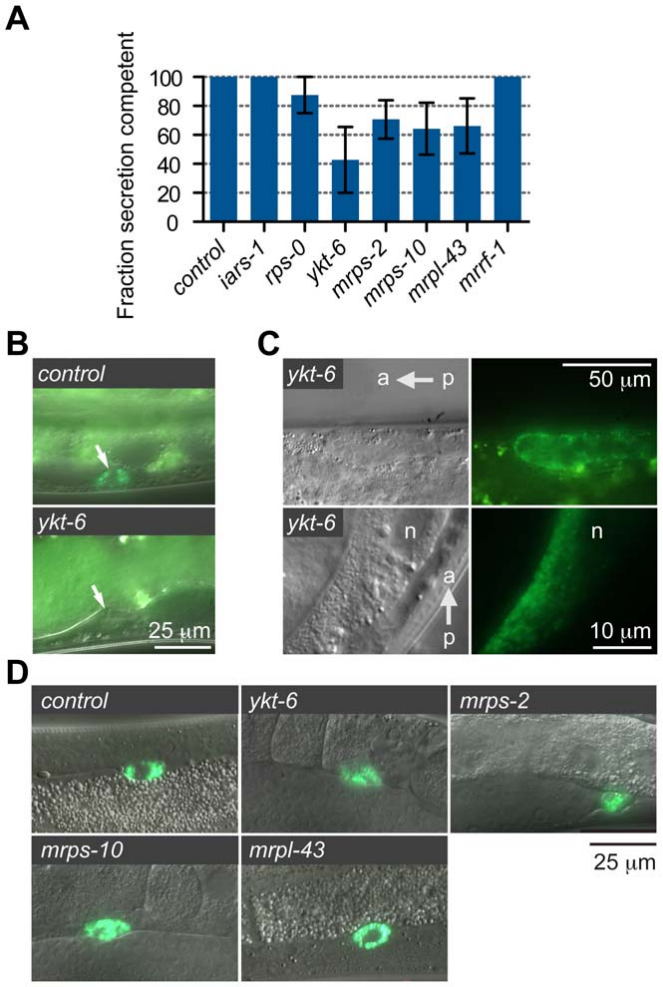
Analysis of DAF-28::GFP and ss::GFP secretion. A) Quantification of DAF-28::GFP secretion defects in control and experimental RNAi worms. Error bars are +/− SE for three individual experiments. B) Paired DIC (top) and fluorescence (bottom) images of *daf-28::gfp* expressing worms exposed to control RNAi (top) or *ykt-6(RNAi)* (bottom). Arrows indicate coelomocytes. No DAF-28::GFP accumulation is seen in the coelomocytes of the *ykt-6(RNAi)* animal shown here. C) Paired DIC (left) and fluorescence (right) images of two *ykt-6*(RNAi) worms that did not secrete *daf-28::gfp*. DAF-28::GFP protein now accumulates around the gonad. n = intestinal nucleus. The anterior/posterior axis is indicated. D) Overlay of DIC and fluorescence images of worms with control RNAi and RNAi against indicated genes to test for the secretion and uptake of ss::GFP. In all cases GFP accumulates only in the coelomocytes (green cells).

To understand the defects that are the basis for the apparent lack of coupling between growth promotion and nutrient availability, we assayed the ability of the knockdown worms to secrete DAF-28/insulin by using a secretion competent DAF-28::GFP reporter encoded by the *svIs69* transgene. In all wild-type animals DAF-28::GFP is secreted into the open circulatory system (the pseudocoelum) and taken up by specialized cells residing there called coelomocytes. Defective DAF-28::GFP secretion is quantified as the percentage of animals that have no GFP labeled coelomocytes [6]. *daf-28* is an agonist for the *daf-2/insulin receptor* and is expressed at high levels only when food is abundant [26], [27]. Secretion of a DAF-28::GFP reporter can be diminished under conditions when worms perceive themselves as being starved [6]. DAF-28::GFP secretion was low in *ykt-6*, *mrps-2*, *mrps-10* and *mrpl-43* knockdown animals (Figure 5A and 5B). Consistent with the finding that *iars-1*, *rps-0* and *mrrf-1* do not influence IIS pathway activity, secretion of DAF-28::GFP in animals was at or near wild-type levels when these genes were inactivated (Figure 5A). The *ykt-6(RNAi)* animals that did not secrete DAF-28::GFP normally displayed a previously unreported DAF-28::GFP accumulation pattern around the gonad (Figure 5C and Figure S1). This accumulation of the reporter protein around the gonad has not been seen in wild-type animals, or other strains that have defects in DAF-28::GFP secretion.

The secretion defects observed here could be caused by a general defect in secretion or a defect in the ability of the coelomocytes to take up secreted proteins. To test if this was the case, we examined the secretion and uptake of a secretion competent GFP encoded by the body muscle expressed *arIs37* transgene. If the GFP is not secreted it will remain in body muscles and not accumulate in the coelomocytes. If coelomocyte function is affected then the GFP will leave the body muscles, but accumulate in the pseudocoelomic space and not the coelomocytes. We observed that secreted GFP was taken up by coelomocytes normally in these RNAi knockdown and control animals (Figure 5D), indicating that coelomocytes are functional and that these animals are secretion competent. We note that due to limitations of the assay system the extent of defective DAF-28::GFP secretion is likely an underestimate of the true extent of the defect, as it can be tested only in adult worms.

## Discussion

Here we report on a screen for new modulators of IIS by analyzing eighty-six genes that encode predicted ASNA-1 interactors and identifying which, upon knocked down, phenocopy the *asna-1* growth phenotype. It is important to note that besides its role in IIS pathway activity, ASNA-1 has several other roles such as the handling of tail-anchored proteins [28], [29] and regulation of metal resistance [30]. Our previous work shows that the IIS activity of ASNA-1 can be genetically separated from its role in metal resistance [31]. The yeast ASNA-1 homolog, Get3, also has roles in G protein signaling and chloride channel function that appear to be independent of its role in the biogenesis of tail-anchored proteins [32], [33]. However we note that for the purpose of this study, this list of genes was used solely as a set of potential modulators of IIS strength. It is possible that some of the genes in this list could modulate other aspects of the ASNA-1 phenotype.

Orthologs of ASNA-1 are highly related across the phylogenetic spectrum and the *Homo sapiens* ortholog can functionally replace the *C. elegans* protein [6], [31]. It was therefore likely that testing the homologs of potential ASNA-1 interacting proteins from different model systems could yield meaningful results. On the other hand if ASNA-1 orthologs have acquired special functions in *S. cerevisiae* or if the genetic interactions in a single celled organism have reduced relevance for a metazoan such an approach can have limited utility. It should be noted that although *S. cerevisiae* does not have an insulin-like signaling pathway, its ASNA-1 ortholog GET3 is responsive to nutritional conditions and plays a role in the adaptation of the yeast cells to different nutritional conditions [34], [35].

Notwithstanding potential drawbacks, this approach was successful in identifying four genes that act in the Golgi network or the mitochondria, are essential for larval growth, are positive modulators of insulin signaling and promote insulin secretion. The screen was also successful in the opposite sense of being able to eliminate potential candidates from further study because of the conditions that were imposed. Two genes of the initial candidate set (*goa-1* and *F41C3.4*) whose RNAi phenotype resembled that of *asna-1*, were eliminated because they had strong feeding defects and two others (*rps-0* and *iars-1*) were also eliminated from further consideration because the larval growth arrest seen in the knockdown animals was not *daf-16* dependent. In *rps-0(RNAi)* animals, DAF-16::GFP was nuclear localized, but since a daf-16 mutant did not suppress growth, the growth phenotype was likely not solely due to lowered IIS.

Knockdown of the Golgi localized v-SNARE *ykt-6* showed that Golgi dysfunction was able to perturb insulin secretion ability. *ykt-6* depletion affects the levels of a Golgi resident protein, and produces a larval arrest phenotype like the one we observed [36]. It is well established that mammalian insulin secretion requires plasma membrane SNARE proteins to promote dense-core vesicle (DCV) docking and release [37] . Here we show that a Golgi SNARE protein also plays a role in insulin secretion. The Golgi network is very elaborate in secretory cells and is the source of secretory vesicles like the dense core vesicles.

DCV biogenesis starts with the active budding of this class of vesicles from the trans-Golgi network (TGN) followed later by acidification of the lumen and removal of mis-incorporated proteins. Timely replenishment of spent DCVs is also in part regulated at the level of TGN function [38]. It is possible that dysfunction of the Golgi via inactivation of *ykt-6* could lead to downstream defects in the formation of secretory vesicles and consequently in DAF-28/insulin secretion. The involvement of a SNARE in worm insulin secretion taken together with previous studies highlighting the importance of the dense-core vesicle component UNC-31/CAPS [39] and the ATPase ASNA-1 in worm insulin secretion shows that the secretion of DAF-28/insulin is mechanistically similar to that of mammalian insulins. The findings using DAF-28 as a model insulin therefore will have general relevance.

At the levels of *ykt-6* inactivation obtained under our experimental conditions, the suppression of the larval growth phenotype by *daf-16* (Figure 3A), nuclear localization of DAF-16::GFP (Figure 3B) and the inhibition of DAF-28::GFP secretion (Figure 5A, 5B) with no effect on ss::GFP secretion (Figure 5D), strongly supports the notion that down-regulation of *ykt-6* leads to defects in IIS strength. We note that suppression of the growth defect of *ykt-6(RNAi)* animals by the *daf-16(mgDf50)* mutation is not complete, likely reflecting the fact that the growth defect is not solely due to down-regulation of IIS. The phenotype seen in *ykt-6 (RNAi)* animals is different from that seen upon RNAi against the mitochondrial ribosomal encoding genes in that dauers or semi-dauers were not seen in the progeny of *ykt-6*(RNAi) animals. One reason for this difference may be that the growth defect imposed by *ykt-6(RNAi)* prevents the affected larvae from reaching the dauer commitment (L2D larva) stage. Another reason for the difference in phenotypes may be because the levels of inactivation by RNAi vary from gene to gene . To our knowledge this is the first report of Golgi dysfunction causing a defect in IIS strength.

For both the Golgi localized v-SNARE protein YKT-6 [36] and for the mitochondrial ribosomal proteins *mrps-2*, *mrps-10* and *mrpl-43* the analysis presented here suggests that growth control was at least partly due to a weaker insulin signaling. This reduction could at least in part be explained as a result of lowered DAF-28/insulin secretion. Since expression of the DAF-28::GFP appeared to be at similar levels in control and experimental RNAi animals, these results suggest that DAF-28 activity can be modulated at the level of secretion which is consistent with previous work [6]. This result reinforces the notion that in addition to the transcriptional regulation of *daf-28* [26], mechanisms regulating DAF-28 activity at other levels remain to be discovered [21].

The reduced IIS activity after RNAi against *mrps-2*, *mrps-10* and *mrpl-43* is likely due to mitochondrial dysfunction. Down-regulation of these genes triggers mitochondrial stress response, as measured by increased *hsp-6::gfp* expression, and there is a decrease in the mitochondrial proton gradient in the RNAi animals. The fact that down-regulation of several mitochondrial ribosomal protein genes produce the same phenotype implies that this effect is likely to be observed for many components of the mitochondrial ribosome.

RNAi against *mrps-2*, *mrps-10* and *mrpl-43* induced the formation of complete dauers (the Daf-c phenotype) under conditions that normally prevent this from happening. For the Daf-c phenotype, the RNAi treatments were synergistic with a weak mutation in a TGF*β* pathway component but not with a weak mutation in the IIS pathway (Figure 3E), suggesting that the phenotype is caused specifically by reduction in IIS pathway activity. This conclusion is supported by the fact that larval growth arrest was *daf-16* dependent and that DAF-28::GFP secretion was low. One attractive explanation for low IIS activity is that the inhibition of ribosomal activity leads to defects in oxidative phosphorylation and redox balance [40] as evidenced by the weaker TMRE staining (Figure 4B). This is important for the mitochondrial ATP/ADP ratio which, when it is high promotes insulin secretion. The findings here are consistent with the energy sensor hypothesis that proposes that defects in energy levels produce specific developmental defects [41] and [42]. However, the demonstration that alteration of mitochondrial activity in one tissue can have an effect in a distant tissue [43] makes it difficult to say whether the observed effect was due to perturbation of mitochondrial activity directly in the DAF-28 secreting cells.

We have previously shown that the onset of mitochondrial dysfunction by inactivation of *tomm-40* causes reduced DAF-28::GFP secretion and consequently weaker IIS signaling. The effect of *tomm-40(RNAi)* on IIS strength could be reversed by over-expressing *daf-28*. [21]. The analysis of *misc-1*also shows the involvement of mitochondrial function in DAF-28 secretion. [44]. The findings presented here reinforce the conclusions from these previous studies that mitochondrial activity is important for insulin secretion and IIS activity likely because of its effect on metabolic activity. This is in keeping with the observations in mouse models and human patients that mitochondrial activity is important for insulin secretion and the diabetic phenotype.

The work presented here emphasizes the role of the mitochondria and the Golgi apparatus in the maintenance of IIS activity. Since the genes identified here are conserved throughout evolution, it is likely that their orthologs will modulate IIS levels in other animals.

## Supporting Information

Figure S1 **Accumulation of DAF-28:GFP in gonadal sheath cells.** Paired DIC (left) and fluorescence (right) images of a *daf-28::gfp; ykt-6(RNAi)* animal showing accumulation of GFP in a honeycomb pattern characteristic of gonadal sheath cells. The DIC image shows that the plane of focus is at the surface of the gonad.
(TIF)

Figure S2 **GST-4::GFP is not up-regulated in the mitochondrial dysfunction mutants.** Fluorescence micrographs of RNAi-treated animals expressing the oxidative stress marker P*gst-4*::GFP::NLS. Five animals are displayed in each panel.
(TIF)

Table S1 **List of genes which are predicted or proven interactors of** ***C.elegans asna-1*** **or its orthologs in** ***S.cerevisiae*** **and** ***D.melanogaster*** **.** The Feeding RNAi screen for the 86 *C. elegans* homologs of total 143 was carried out and following phenotypes are listed in the last column. Wt: Wildtype; Lva: larval arrest; Sck: Sick; Prl: paralysed Let: lethal; Emb: Embryonic Lethal; Gro: Slow growing; Ste: Sterile.
(DOC)

Table S2 **Mitoprot analysis of putative** ***C. elegans*** **mitochondrial ribosomal proteins.**
(DOC)
