# Supplementary figures and images for: A Directed RNAi Screen Based on Larval Growth Arrest Reveals New Modifiers of C. elegans Insulin Signaling
Source: PLoS One. 2012 Apr 12;7(4):e34507. doi: 10.1371/journal.pone.0034507 (PMC3325266; doi:10.1371/journal.pone.0034507)

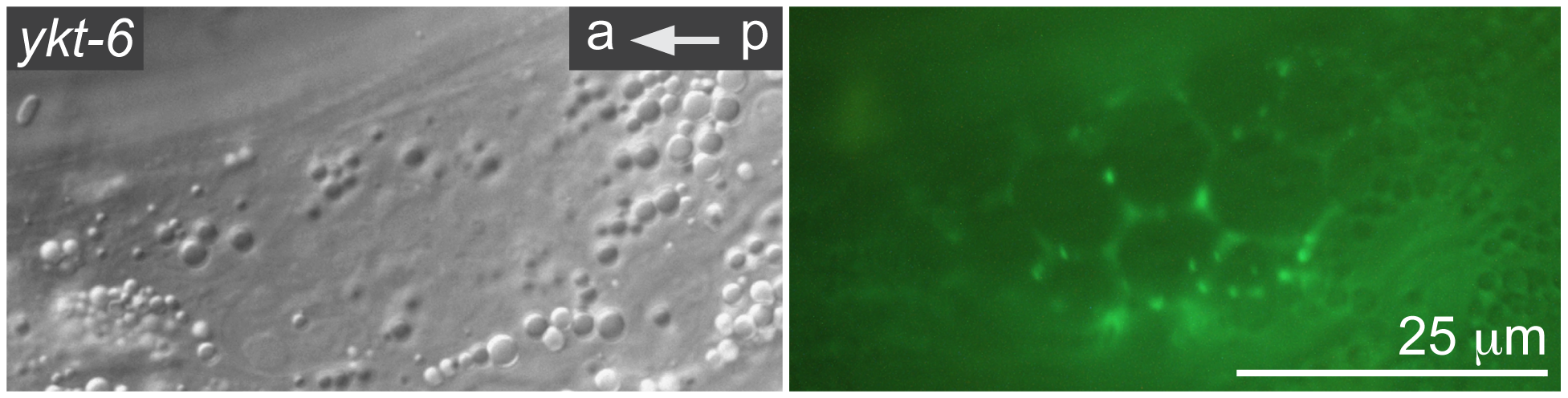

Supplement: Figure S1 — Accumulation of DAF-28:GFP in gonadal sheath cells. Paired DIC (left) and fluorescence (right) images of a daf-28::gfp; ykt-6(RNAi) animal showing accumulation of GFP in a honeycomb pattern characteristic of gonadal sheath cells. The DIC image shows that the plane of focus is at the surface of the gonad. (TIF) [file pone.0034507.s001.tif]

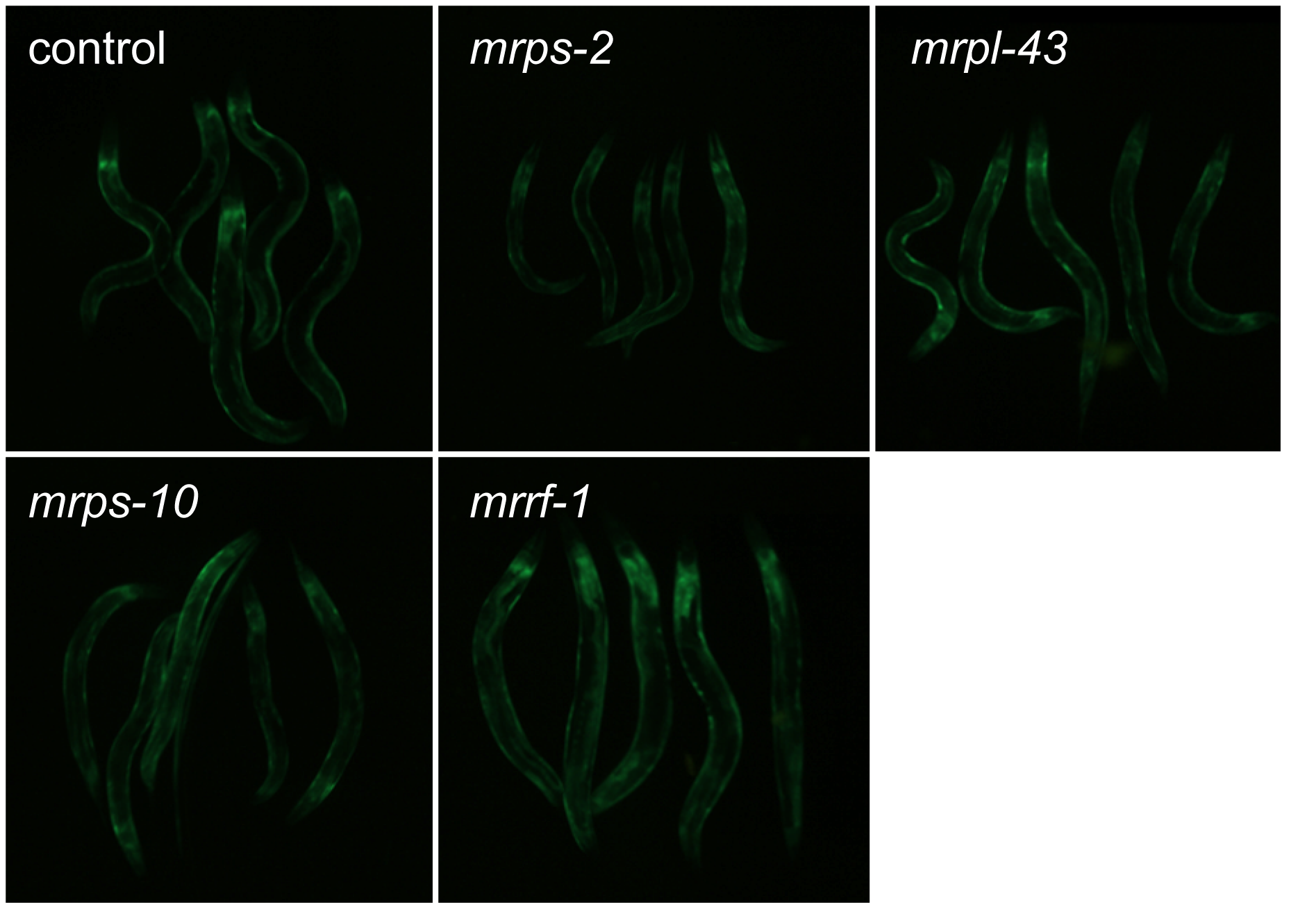

Supplement: Figure S2 — GST-4::GFP is not up-regulated in the mitochondrial dysfunction mutants. Fluorescence micrographs of RNAi-treated animals expressing the oxidative stress marker Pgst-4::GFP::NLS. Five animals are displayed in each panel. (TIF) [file pone.0034507.s002.tif]
