# Supplementary material for: A Directed RNAi Screen Based on Larval Growth Arrest Reveals New Modifiers of C. elegans Insulin Signaling
Source: PLoS One. 2012 Apr 12;7(4):e34507. doi: 10.1371/journal.pone.0034507 (PMC3325266; doi:10.1371/journal.pone.0034507)
Supplement: Table S1 — List of genes which are predicted or proven interactors of C.elegans asna-1 or its orthologs in S.cerevisiae and D.melanogaster . The Feeding RNAi screen for the 86 C. elegans homologs of total 143 was carried out and following phenotypes are listed in the last column. Wt: Wildtype; Lva: larval arrest; Sck: Sick; Prl: paralysed Let: lethal; Emb: Embryonic Lethal; Gro: Slow growing; Ste: Sterile. (DOC) [file pone.0034507.s003.doc]

Table S1.

List of the genes that are predicted or proven interactors of *C.elegans* *asna-1* or its orthologs in *S.cerevisiae* and *D.melanogaster*.

| ***Interactor*** | ***(Reference for previously found interaction)*** | ***Corresponding C. elegans gene*** | ***BLAST***  ***E-value*** | ***C. elegans phenotype*** |
| --- | --- | --- | --- | --- |
| ***To yeast Get3*** |  |  |  |  |
| APL5 | (Schuldiner et. al., 2005) | T20B5.1 | 1.1e-19 | Prl, |
| APL5 | (Schuldiner et. al., 2005) | W09G10.4 | 7.0e-97 | Wt |
| ARF2 | (Schuldiner et. al., 2005) | Y116A8C.12 | 7.5e-60 | Wt |
| ARL1 | (Tong et. al., 2004) | F54C9.10 | 2.7e-55 | Emb |
| ARL1, ARF2 | (Tong et. al., 2004, Schuldiner et. al., 2005) | B0336.2 | 2.4e-54, 1.2e-77 | Sck, Let, Ste |
| ARL1, ARF2 | (Tong et. al., 2004, Schuldiner et. al., 2005) | F57H12.1 | 5.8e-53, 2.0e-73 | Lva, Gro |
| ARV1 | (Schuldiner et. al., 2005) | R05H5.5 | 2.3e-12 | Wt |
| BAT1 | (Ho et. al., 2002) | K02A4.1 | 1.0e-82 | Wt |
| BAT2 | (Ho et. al., 2002) | Y44A6D.5 | 2.0e-64 | Wt |
| BFR1 | (Schuldiner et. al., 2005) | F01G10.5 | 4.2e-11 |  |
| BRE5 | (Schuldiner et. al., 2005) | C09D1.1 | 4.8e-08* |  |
| BZZ1 | (Ito et. al., 2001) | F09E10.8 | 1.8e-13 |  |
| COG5 | (Schuldiner et. al., 2005) | C17H1.4 | 9.0e-04* |  |
| COG6 | (Schuldiner et. al., 2005) | K07C11.9 | 3.9e-09 | Wt |
| COG7 | (Schuldiner et. al., 2005) | F56D5.9 | 0.0014 |  |
| COG8 | (Schuldiner et. al., 2005) | F36H12.3 | 2.8e-15 |  |
| CPR2 | (Ito et. al., 2001) | C34D4.12 | 5.7e-30 | Wt |
| CPR2 | (Ito et. al., 2001) | D1009.2 | 1.4e-40 | Wt |
| CPR2 | (Ito et. al., 2001) | F42G9.2 | 7.6e-51 | Wt |
| CPR2 | (Ito et. al., 2001) | F59E10.2 | 9.4e-27 | Wt |
| CPR2 | (Ito et. al., 2001) | T01B7.4 | 9.1e-39 | Wt |
| CPR2 | (Ito et. al., 2001) | T27D1.1 | 3.6e-37 | Wt |
| CPR2 | (Ito et. al., 2001) | Y75B12B.2 | 3.8e-40 |  |
| CPR2 | (Ito et. al., 2001) | Y75B12B.5 | 4.6e-44 | Wt |
| CPR2 | (Ito et. al., 2001) | ZK520.5 | 6.9e-41 | Wt |
| CRH1 | (Ito et. al., 2001) | H02F09.3 | 1.2e-21* |  |
| CUE4 | (Ito et. al., 2001) | F44B9.5 | ** |  |
| DPM1 | (Schuldiner et. al., 2005) | H43I07.3† | 2.5e-04 |  |
| ERG25 | (Schuldiner et. al., 2005) | F35C8.5 | 2.8e-21 | Wt |
| ERG25 | (Schuldiner et. al., 2005) | F49E12.10 | 7.6e-19 | Wt |
| ERG25 | (Schuldiner et. al., 2005) | F49E12.9 | 9.2e-23 | Wt |
| ERV14 | (Schuldiner et. al., 2005) | T09E8.3 | 1.1e-17 | Wt |
| ERV2 | (Ito et. al., 2001) | F56C11.3 | 2.6e-18 | Wt |
| FUR1 | (Ito et. al., 2001) | C47B2.2 | 2.6e-34* |  |
| GCR2 | (Ito et. al., 2001) | H02F09.3 | 6.7e-05* |  |
| GIM4 | (Schuldiner et. al., 2005) | H20J04.5† | 7.0e-09 |  |
| GLO3 | (Schuldiner et. al., 2005) | F07F6.4 | 1.9e-30 | Wt |
| GLO3 | (Schuldiner et. al., 2005) | K02B12.7 | 3.1e-18 | Wt |
| GLO3 | (Schuldiner et. al., 2005) | W09D10.1 | 9.2e-13 | Wt |
| GOT1 | (Schuldiner et. al., 2005) | F41C3.4 | 1.3e-23 | Lva |
| GPA1 | (Ito et. al., 2001) | B0207.3 | 2.7e-40 | Wt |
| GPA1 | (Ito et. al., 2001) | C16A11.1 | 8.9e-54 | Wt |
| GPA1 | (Ito et. al., 2001) | C26C6.2 | 8.4e-84 | Lva |
| GPA1 | (Ito et. al., 2001) | C34D1.3 | 6.9e-74 | Wt |
| GPA1 | (Ito et. al., 2001) | C55H1.2 | 7.6e-51 | Wt |
| GPA1 | (Ito et. al., 2001) | E02C12.5 | 1.1e-75 | Wt |
| GPA1 | (Ito et. al., 2001) | F18E2.5 | 6.2e-51 | Wt |
| GPA1 | (Ito et. al., 2001) | F18G5.3 | 2.4e-59 | Wt |
| GPA1 | (Ito et. al., 2001) | F38E1.5 | 7.4e-53 | Wt |
| GPA1 | (Ito et. al., 2001) | F48C11.1 | 3.4e-58 | Wt |
| GPA1 | (Ito et. al., 2001) | F56H9.3 | 2.7e-60 | Wt |
| GPA1 | (Ito et. al., 2001) | F56H9.4 | 8.8e-48 | Wt |
| GPA1 | (Ito et. al., 2001) | M01D7.7 | 3.0e-59 | Wt |
| GPA1 | (Ito et. al., 2001) | M04C7.1 | 3.4e-62 | Wt |
| GPA1 | (Ito et. al., 2001) | R06A10.2 | 9.2e-57 | Wt |
| GPA1 | (Ito et. al., 2001) | R10H10.5 | 4.1e-80 | Wt |
| GPA1 | (Ito et. al., 2001) | T07A9.7 | 2.0e-78 | Wt |
| GPA1 | (Ito et. al., 2001) | T19C4.6 | 5.4e-70 | Wt |
| GSF2 | (Ho et. al., 2002) | - |  |  |
| GSG1 | (Schuldiner et. al., 2005) | - |  |  |
| HAC1 | (Schuldiner et. al., 2005) | Y41C4A.4† | ** |  |
| HSD1 | (Schuldiner et. al., 2005) | C33H5.18 | ** |  |
| HSP82 | (Zhao et. al., 2005) | R151.7 | 1.5e-90 | Wt |
| HSP82 | (Zhao et. al., 2005) | T05E11.3 | 2.4e-166 | Lva |
| ILS1 | (Gavin et. al., 2002) | C25A1.7 | 2.0e-60 | Gro |
| ILS1 | (Gavin et. al., 2002) | R11A8.6 | 0 | Lva |
| INO1 | (Ito et. al., 2001) | VF13D12L.1 | 1.5e-134* |  |
| MDH2 | (Ito et. al., 2001) | F20H11.3 | 5.5e-54 | Emb, Gro |
| MDH2 | (Ito et. al., 2001) | F46E10.10 | 5.8e-06 | Wt |
| MDM39 | (Krogan et. al., 2006) | - |  |  |
| MGA2 | (Schuldiner et. al., 2005) | B0350.2 | 3.0e-05* |  |
| MRP4 | (Ito et. al., 2001) | B0393.1 | 1.7e-09 | Lva |
| MRP4 | (Ito et. al., 2001) | T23B12.3 | 8.4e-21 | Lva |
| MSN4 | (Ho et. al., 2002) | C55B7.12 | 2.2e-10 | Wt |
| NNF2 | (Schuldiner et. al., 2005) | M151.4 | 3.7e-07* |  |
| OPI3 | (Schuldiner et. al., 2005) | - |  |  |
| PHO90 | (Schuldiner et. al., 2005) | F31F6.6 | 5.3e-19* |  |
| PMR1 | (Schuldiner et. al., 2005) | ZK256.1† | 3.5e-165 |  |
| POR1 | (Ho et. al., 2002) | R05G6.7 | 2.8e-21 | Wt |
| PSK1 | (Krogan et. al., 2006) | H39E23.1 | 3.6e-29* |  |
| RGP1 | (Schuldiner et. al., 2005) | - |  |  |
| RIC1 | (Schuldiner et. al., 2005) | - |  |  |
| RMD7 | (Krogan et. al., 2006) | - |  |  |
| RPN4 | (Pan et. al., 2006) | C55B7.12 | 2.2e-10 |  |
| RUD3 | (Schuldiner et. al., 2005) | C18C4.5 | 5.6e-17* |  |
| SBH2 | (Schuldiner et. al., 2005) | Y38F2AR.9† | 4.0e-06 |  |
| SCS2 | (Schuldiner et. al., 2005) | F33D11.11 | 8.4e-11 | Wt |
| SCS2 | (Schuldiner et. al., 2005) | F42G2.5 | 1.6e-10 | Wt |
| SEC22 | (Schuldiner et. al., 2005) | B0361.10 | 2.8e-07 | Lva |
| SEC22 | (Schuldiner et. al., 2005) | F55A4.1 | 1.1e-28 | Wt |
| SEC27 | (Schuldiner et. al., 2005) | Y71F9AL.17† | 1.4e-61 |  |
| SEC28 | (Schuldiner et. al., 2005) | F45G2.4 | ** |  |
| SEC66 | (Schuldiner et. al., 2005) | - |  |  |
| SEL1 | (Schuldiner et. al., 2005) | ZK353.8 | ** | Wt |
| SKG1 | (Ito et. al., 2001) | - |  |  |
| SPF1 | (Schuldiner et. al., 2005) | C10C6.6 | 2.2e-223 | Wt |
| SPF1 | (Schuldiner et. al., 2005) | K07E3.7 | 1.4e-88 |  |
| SRP72 | (Schuldiner et. al., 2005) | F08D12.1 | 1.9e-19* |  |
| SSH1 | (Schuldiner et. al., 2005) | Y57G11C.15† | 4.9e-63 |  |
| SUC2 | (Ito et. al., 2001) | - |  |  |
| SWA2 | (Schuldiner et. al., 2005) | W07A8.3 | 7.8e-07* |  |
| SYS1 | (Schuldiner et. al., 2005) | T03F1.12† | 4.2e-11* |  |
| TAT1 | (Krogan et. al., 2006) | C50D2.2 | 2.6e-13 | Wt |
| TAT1 | (Krogan et. al., 2006) | F07C3.7 | 9.6e-09 |  |
| TLG2 | (Schuldiner et. al., 2005) | F55A11.2 | 1.2e-09 | Emb |
| TLG2 | (Schuldiner et. al., 2005) | ZC155.7 | 3.7e-20 | Wt |
| YAH1 | (Ito et. al., 2001) | F55B11.1 | ** | Sck |
| YAP6 | (Ito et. al., 2001) | ZC308.1 | 1.2e-05 |  |
| YAP6 | (Ito et. al., 2001) | T27F2.4 | ** |  |
| YBR014C | (Ho et. al., 2002) | F10D7.3 | 5.0e-15 | Wt |
| YBR255W | (Schuldiner et. al., 2005) | ZK131.11 | 1.4e-05* |  |
| YDL121C | (Ito et. al., 2001) | T24H7.2 | 4.7e-3 |  |
| YDR415C | (Ito et. al., 2001) | C35C5.2 | 1.2e-4 | Wt |
| YDR415C | (Ito et. al., 2001) | R57.1 | 5.0e-3 | Wt |
| YHP1 | (Krogan et. al., 2006) | C09G12.1 | 4.1e-07 | Wt |
| YHP1 | (Krogan et. al., 2006) | ZC64.4 | 4.5e-06 | Wt |
| YHP1 | (Krogan et. al., 2006) | ZK993.1† | 4.0e-09 |  |
| YJL123C | (Schuldiner et. al., 2005) | C35E7.9 | 5.0e-08 |  |
| YOL111C | (Ito et. al., 2001) | ZK688.5 | ** | Wt |
| YOL159C | (Ito et. al., 2001) | - |  |  |
| YOR164C | (Ito et. al., 2001) | F26F4.1 | 3.6e-04 | Wt |
| YPT6 | (Tong et. al., 2004) | F26H9.6 | 8.2e-38 | Emb |
| YPT6 | (Tong et. al., 2004) | F59B2.7 | 3.8e-65 | Wt |
| YPT6 | (Tong et. al., 2004) | T25G12.4 | 1.1e-63 | Wt |
| ***To Drosophila CG1598*** |  |  |  |  |
| CG11382 | (Giot et. al., 2003) | Y39B6A.1† | 6.0e-04* |  |
| CG3838 | (Giot et. al., 2003) | T07C12.11 | 1.0e-3* |  |
| CG6357 | (Giot et. al., 2003) | Y40H7A.10† | 8.0e-07* |  |
| CG13394 | (Giot et. al., 2003) | - |  |  |
| CG8603 | (Giot et. al., 2003) | T09A5.10 | 9.0e-05* |  |
| CG9614 | (Giot et. al., 2003) | C34F6.4 | 1.0e-19 | Wt |
| ***To C. elegans asna-1*** |  |  |  |  |
| C18E9.6 | (Zhong et. al., 2006) | C18E9.6 |  | Lva, Ste, Gro |
| D2045.6 | (Li et. al., 2004) | D2045.6 |  | Wt |
| F08C6.6 | (Li et. al., 2004) | F08C6.6 |  | Wt |
| F26F4.1 | (Li et. al., 2004) | F26F4.1 |  | Wt |
| F40H3.2 | (Li et. al., 2004) | F40H3.2 |  | Wt |
| F52B5.1 | (Zhong et. al., 2006) | F52B5.1 |  | Wt |
| M01H9.2 | (Li et. al., 2004) | M01H9.2† |  |  |
| R05G6.7 | (Zhong et. al., 2006) | R05G6.7 |  | Wt |
| R09B5.5 | (Li et. al., 2004) | R09B5.5 |  | Wt |
| Y71G12B.27 | (Li et. al., 2004) | Y71G12B.27† |  |  |
| ZC434.8 | (Li et. al., 2004) | ZC434.8† |  |  |
| F44B9.6 | (Li et. al., 2004) | F44B9.6 |  | Wt |
| T21B6.3 | (Walhout et. al., 2000) | T21B6.3 |  | Wt |

The Feeding RNAi screen for the 86 *C. elegans* homologs of total 143 was carried out and following phenotypes are listed in the last column.

Wt: Wildtype; Lva: larval arrest; Sck: Sick; Prl: paralysed Let: lethal; Emb: Embryonic Lethal; Gro: Slow growing; Ste: Sterile.

(†): Clone not available in RNAi library.

(*): Identified using only SMART

(**): Identified using only BLAST.

References

1. M. Schuldiner *et al.*, *Cell* **123**, 507 (Nov 4, 2005).

2. A. H. Tong *et al.*, *Science* **303**, 808 (Feb 6, 2004).

3. Y. Ho *et al.*, *Nature* **415**, 180 (Jan 10, 2002).

4. T. Ito *et al.*, *Proc Natl Acad Sci U S A* **98**, 4569 (Apr 10, 2001).

5. R. Zhao *et al.*, *Cell* **120**, 715 (Mar 11, 2005).

6. A. C. Gavin *et al.*, *Nature* **415**, 141 (Jan 10, 2002).

7. N. J. Krogan *et al.*, *Nature* **440**, 637 (Mar 30, 2006).

8. Y. Pan, A. Grindstaff, D. Cassada, M. Goldman, J. Taylor, *Transplantation* **63**, 1032 (Apr 15, 1997).

9. L. Giot *et al.*, *Science* **302**, 1727 (Dec 5, 2003)

10. S. Li *et al.*, *Science* **303**, 540 (Jan 23, 2004).

11. W. Zhong, P. W. Sternberg, *Science* **311**, 1481 (Mar 10, 2006).

12. A. J. Walhout *et al.*, *Science* **287**, 116 (Jan 7, 2000)
