# Supplementary material for: A Directed RNAi Screen Based on Larval Growth Arrest Reveals New Modifiers of C. elegans Insulin Signaling
Source: PLoS One. 2012 Apr 12;7(4):e34507. doi: 10.1371/journal.pone.0034507 (PMC3325266; doi:10.1371/journal.pone.0034507)
Supplement: Table S2 — Mitoprot analysis of putative C. elegans mitochondrial ribosomal proteins. (DOC) [file pone.0034507.s004.doc]

Table S2: Mitoprot analysis of putative *C. elegans* mitochondrial ribosomal proteins

| ***Protein*** | ***Cleaved sequence*** | ***Probability of export to mitochondria*** |
| --- | --- | --- |
| MRPS-2 | MRRVVAAAIRTERAFSTRFSAS | 0.87 |
| MRPS-10 | MLKLASSLRTGLISRSIRTLAPTVNPA | 0.98 |
| MRPL-43 | MPSVPRVDRLKPIYTAAKALNFGWRFSDFLKIPAYNGISRYTNQLHRI | 0.94 |
| MRRF-1 | Not predictable | - |
